# Dr. J. G. Westmoreland on “Actions of Mercury”

**Published:** 1875-04

**Authors:** 


The positions taken by Dr. J. G. Westmoreland in his essay read before the Academy of Medicine on the "Actions of Mercury," were incorrectly reported in the last number of our Journal. He is reported as saying he "objected to the results of experiments made, with reference to the effect of mercury, on healthy dogs." He does not object to the results nor deny their correctness, but thinks the conclusions arrived at probably erroneous. Again, he is made to say, "he objected to a classification based on the action of remedies," while his paper shows he approved of a classification based exclusively on the direct, primary or physiological action of remedies.

